# Temperature‐Dependent Warning Colour Variation in an Aposematic Insect

**DOI:** 10.1002/ece3.73683

**Published:** 2026-11-10

**Authors:** Jiajia Zhao, Daniella Black, Madingley M. Abbs, Terry K. Smith, Sally L. Shirran, Silvia A. Synowsky, Hannah M. Rowland, Astrid T. Groot, David M. Shuker, Emily R. Burdfield‐Steel

**Affiliations:** ^1^ Institute for Biodiversity and Ecosystem Dynamics University of Amsterdam Amsterdam the Netherlands; ^2^ School of Biology University of St Andrews St Andrews UK; ^3^ BSRC Mass Spectrometry & Proteomics Facility University of St Andrews St Andrews UK; ^4^ Department of Evolution, Ecology and Behaviour Institute of Infection, Veterinary and Ecological Sciences, University of Liverpool Liverpool UK

**Keywords:** aposematic colour, polymorphism, proteomics, temperature

## Abstract

Aposematic colouration in animals advertises prey toxicity and unpalatability to predators. However, intra‐specific variation in aposematic signals is widespread and often thought to be the result of environmental factors such as temperature. To investigate how temperature affects warning colour, we used the aposematic seed bug *Lygaeus simulans* as a model. Wild‐type adults of this species exhibit the typical red and black pattern common among many aposematic insects, while “pale” mutants are yellow and black. Here, we investigated how rearing temperatures affect their warning colour, fitness traits, and protein expression. We found that *L. simulans* warning colouration is temperature sensitive, with both pale‐mutant and wild‐type individuals redder at lower temperatures (24°C) compared to higher (32°C) temperatures. Reduced body size (weight and length) was also found at this lower temperature. Utilising SWATH‐proteomic analysis, we identified 15 differentially expressed proteins between the two morphs, including *Glutathione S‐transferase* (GST), a protein involved in oxidative states. Subsequent analysis of GST activity showed higher activity in pale‐mutants compared to wild‐types at 29°C. Furthermore, in males, this difference between morphs mirrored the colour change as it was not evident at 24°C, although this was not the case in females. Our research reveals a distinctive temperature‐dependent variation in aposematic colouration, providing a greater understanding of how warning signals evolve with temperature changes.

## Graphical abstract

The study investigated the temperature effect on aposematic colour, fitness traits, and protein expression in *Lygaeus simulans*. The aposematic colour in the species is temperature‐dependent, and two morphs show differences in protein expression. The findings demonstrate how aposematic species evolve with temperature changes.

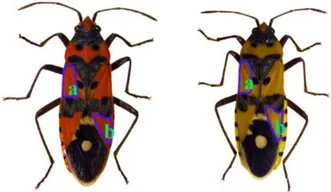

## 1 Introduction

Animals have evolved a variety of defensive strategies against predation, including crypsis, mimicry, and aposematism (Ruxton et al. 2018). Among these, warning colouration is widely used by organisms to signal their toxicity and unpalatability to predators (Arenas et al. 2014; Burdfield‐Steel et al. 2025; Rojas et al. 2015). The effectiveness of conspicuous visual signals is dependent on learning in natural enemies and those enemies' experience of a prey's unprofitability (Roper and Redston 1987; Svádová et al. 2009), which leads to the expectation of uniformity and reduced intraspecific variation in these signals (Speed and Ruxton 2005). However, colour variation can be found within and between many aposematic species. Such colour variation is often shaped by ecological factors, such as temperature and season (Briolat et al. 2019; Nice and Fordyce 2006), as well as shifting predation pressures, or different predator communities (Foster et al. 2023; Rönkä et al. 2020). In addition, human activities, such as deforestation, can shift both abiotic conditions and ecological interactions, leading to selection for colour changes (Ni et al. 2024). Thus, there is an increasing need to investigate aposematic colour variation.

The role of temperature in colour variation has been demonstrated in some insect species, such as Lepidoptera (Briolat et al. 2019) and Odonata (Pinkert et al. 2017). Black colouration in insects typically decreases with increasing temperature (i.e., thermal melanism; Majerus 1998), but there are only a few examples of temperature effects on bright warning colouration (Fabricant et al. 2018). Temperature has been considered as one of the most important factors affecting traits underlying insect fitness (Overgaard and Sørensen 2008), such as body size, development time, and reproduction (Fox and Czesak 1999; Ferrer et al. 2013). On the other hand, colour variation is also likely to have associated trade‐offs with chemical defences (Bezzerides et al. 2007; Blount et al. 2009) or life‐history traits (e.g., growth rate and size; Ojala et al. 2007; Ma et al. 2008), due to the consumption of amino acids (Futahashi and Osanai‐Futahashi 2021) and/or antioxidants (Blount et al. 2009, 2023).

While the ecological, physiological, and genetic basis of colour variation is increasingly well understood (Daniels et al. 2014), the protein‐level mechanisms that generate and maintain such phenotypes—and mediate their interactions with other life history traits—remain comparatively underexplored (Valcu and Kempenaers 2015). Recent advances in mass‐spectrometry (MS)‐based approaches (e.g., SWATH‐MS) have expanded the toolkit for studying proteomics variation in non‐model organisms. SWATH‐MS is an emerging label‐free technique with better coverage than traditional shotgun proteomics (Gillet et al. 2012). This tool has previously been used to identify and quantify proteins in insects such as Asian hornets (Alonso‐Sampedro et al. 2023), flies (Fabre et al. 2019), and silkworms (Hu et al. 2017). For example, Wang et al. (2023) identified 324 proteins expressed differently in two strains (cyan and yellow colour) of the silk moth *Antheraea pernyi*. He et al. (2022) combined RNA‐seq and SWATH‐MS proteomics to identify differentially expressed proteins such as cuticular proteins and chitinases in two morphs of the butterfly *Papilio xuthus* .

In true bugs (order Hemiptera), warning colouration is common, particularly among the Lygaeidae. Approximately 20% of species are aposematic, often with a distinctive black and red pattern (Burdfield‐Steel and Shuker 2014). In the seed bug *Lygaeus simulans*, which has a Palearctic distribution (Balfour et al. 2025) and is widespread across Southern and Central Europe (Burdfield‐Steel et al. 2013; Heyden and Dioli 2018), wild‐type adults display the typical red and black pattern. However, in a laboratory population, a “pale” mutant was discovered that exhibited a yellow‐black colour (Figure S1; Balfour et al. 2018). The colour difference can also be seen in nymphs, which are red and white in wild‐type individuals, but grey‐green in the pale mutant. The pale phenotype is inherited in a strictly Mendelian pattern, with the pale allele being recessive (Balfour et al. 2018, 2020). Observational evidence suggests that the pale mutant is also temperature‐sensitive, with individuals exposed to lower temperatures resembling the wild‐type in colour (Abbs, pers. obvs), but the timing and cause of this switch are unknown.

Here, we analysed adult colour variation to test the hypothesis that the warning colouration in *L. simulans* is temperature‐dependent. We predicted that individuals reared at lower temperatures would exhibit increased red colouration, and that this response would be more pronounced in the pale‐mutant. We further predicted that the colour changes may be associated with physiological trade‐offs and differences in protein expression, particularly in pathways related to oxidative stress such as *Glutathione S‐transferase*.

## 2 Materials and Methods

### 2.1 Insect Rearing

The pale‐mutant and wild‐type lines were reared at the University of St Andrews and the University of Amsterdam. The Amsterdam laboratory strains that we used in the temperature experiments were obtained from the Shuker laboratory at the University of St Andrews. This population originated from individuals collected from the wild in Tuscany, Italy in 2008 and 2009. They were kept in climate cabinets at 29°C with 22:2 h (light: dark) and 60% humidity to prevent diapause.

In the temperature experiments, pairs of males and females were separately reared in Petri dishes at 29°C and eggs were collected every day. We collected either (a) 1‐day old eggs or (b) second instar nymphs daily and transferred them into 24°C (low), 26°C (medium), 29 C (control) and 32°C (high) temperature climate chambers with 22 L: 2D and 60% relative humidity. Once the bugs reached adulthood, they were used for the subsequent measurements. Bug samples for proteomics were collected at the University of St Andrews, and full details can be found in Methods S1.

### 2.2 Impact of Temperature on Colour Polymorphism

Adults were frozen at −20°C for 1 day. Photos were taken in a dark room with a sunray lamp above the bugs to ensure standardised lighting conditions. Adults (*n* = 40) were pinned to a polypropylene background with a grey standard (40 mm × 40 mm) next to it. To take photos of the bugs, first with a visible and then with an ultraviolet (UV) camera filter, a Samsung camera (NX1000) was used, customised into full spectrum range and equipped with a Novoflex Noflexar 35 mm lens. The visible‐filter photos were taken with an ISO of 200 and the UV‐filter photos were taken with an ISO of 12,800, with low, medium, and high exposure.

We analysed the colours using good quality images (those that were in focus and not overexposed) and the micaToolbox (Troscianko and Stevens 2015). Visible and UV photos were combined to create multispectral images. The grey standard was used to normalise the standard reflectance and as a scale bar. Two aposematic colour regions of interest in adults (Figure S1) were chosen and both regions were used for subsequent analysis. Then, generated files were used to create cone‐catch values in human vision. Finally, a three‐dimensional colour space CIELAB (CIE L*a*b*) (Zhang and Wandell 1997) was generated. In this space, colour was described by luminance: a Red‐Green channel, where higher positive values signify a shift towards red; and a Yellow‐Blue channel, where higher positive values indicate a shift towards yellow (see Figure 1; McGuire 1992).

**FIGURE 1:**
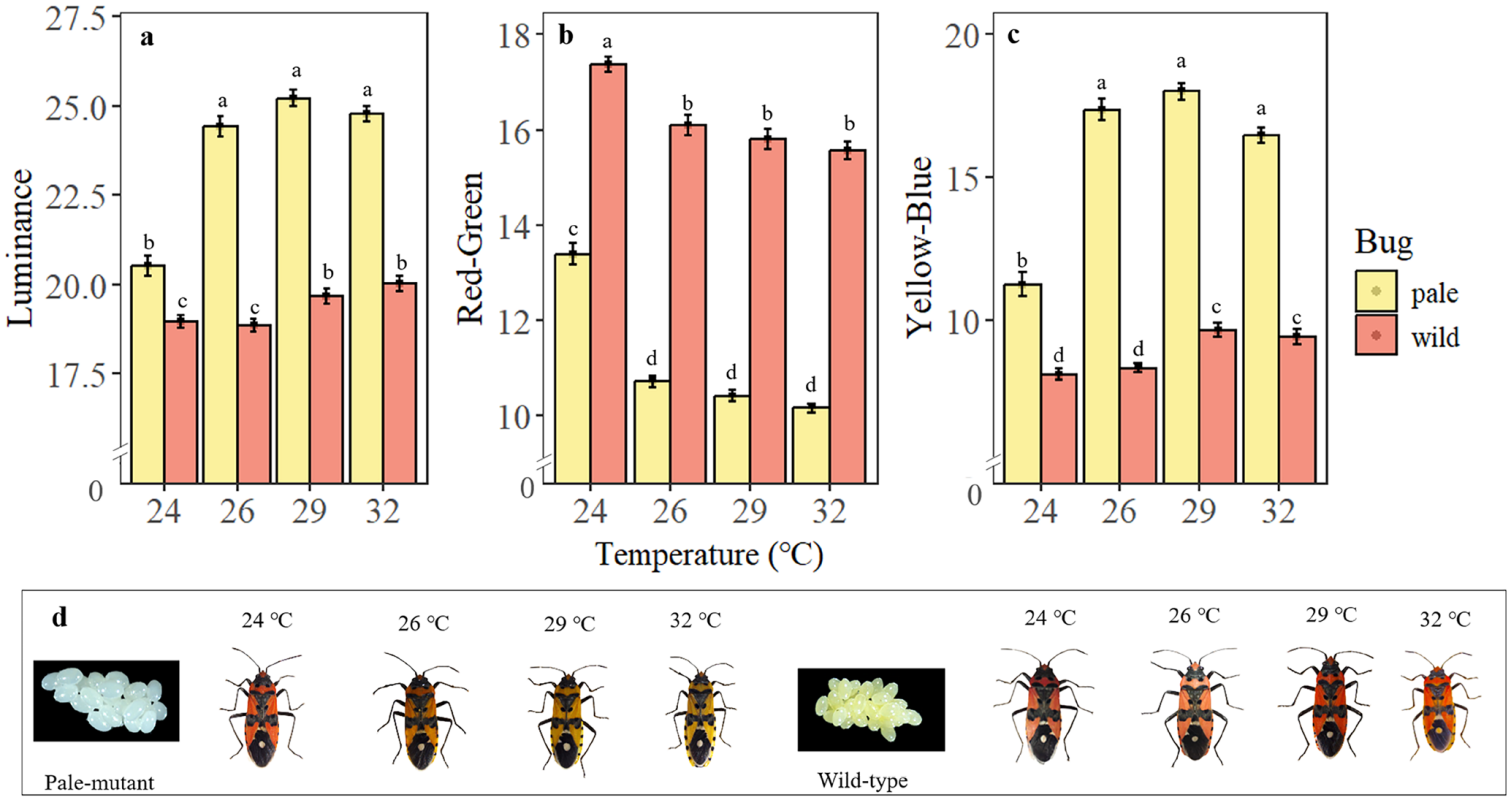
Warning colour signals of adults reared at different temperatures (*N* = 320). Warning colour was estimated by (a) Luminance, (b) Red‐Green opponent channels and (c) Yellow‐Blue opponent channels. Pale‐mutants are shown in yellow and wild‐types are shown in red. The X‐axis represents the different temperatures (24°C, 26°C, 29°C and 32°C). (d) Aposematic colour of bugs reared at different temperatures. The adults in the above figure were transferred to the temperatures at the egg stage. Bars represent means of colour value ±SEs. Letters represent significant differences in Tukey's post hoc tests.

### 2.3 Consequences of Temperature on Colour Polymorphism

Newly moulted adults reared under low, medium, and high temperatures were collected daily for survival (*n* = 5, see details in Methods S1), weight, and length assays. The sample sizes of the weight and length data in each treatment can be found in the File S1. Body length measurements of males and females (see Figure S2) were performed separately under a microscope (Zeiss Stemi 508) with 10 mm × 10 mm grid paper.

### 2.4 Proteomics Analysis

To measure proteome expression of *L. simulans* pale‐mutant and wild‐type morphs, SWATH‐MS analysis was performed on the whole body (*n* = 3; per phenotype and sex combination) of reproductively mature virgin males and females reared at 29°C. Protein extraction, identification, and data‐independent acquisition were performed as described in the Methods S1. Briefly, adult proteins were extracted using ammonium bicarbonate and protein concentration was measured using the Pierce BCA protein assay kit. IDA library of protein identification and data‐independent acquisition were performed by nanoLC MS/MS analysis (IDA‐LC–MS/MS).

### 2.5 GST Enzymatic Activity Assays

Total GST enzymatic activity was measured in virgin males and females using *Glutathione S‐transferase* assay kits (Sigma‐Aldrich, MAK 453). This kit is based on the conjugation of reduced glutathione (GSH) to 1‐chloro‐2,4‐dinitrobenzene (CDNB), a standard substrate. GST enzymes catalyse this reaction, and the formation of the GSH‐CDNB conjugate is detected by absorbance. The protocol followed the kit manual. Individuals (*n* = 9; per phenotype, sex and temperature combination) were flash‐frozen in liquid nitrogen. They were then homogenised using TissueLyser for 90 s at a speed of 28 1/s in 250 μL buffer containing 100 mM potassium phosphate buffer and 2 mM EDTA (pH = 7). Tubes were centrifuged briefly to transfer all homogenate. Samples were frozen at −80°C for 5 min to lyse cells and then centrifuged at 10,000 rpm at 4°C. Clean supernatants were prepared for subsequent assays. 20 μL diluted samples (1:1; v/v) and 180 μL working reagents were prepared for analysis. Density of 340 nm was measured in kinetic mode at 25°C using SPECTROstar Nano (BMG Labtech).

### 2.6 Data Analysis

Data analysis and visualisation were performed using R studio statistical software (R Core Team 2022). Aposematic colour was measured as Luminance, the Red‐Green opponent channel, and the Blue‐Yellow opponent channel. The average across the two regions of interest for each individual was calculated and the data were then analysed by linear models that included morph (pale‐mutant or wild‐type) and temperature, as well as two‐way interactions between temperature and morph. Tukey's post hoc tests were then performed to compare effects across all treatments. For weight and length data, the linear models were similar to the above but contained the additional factor of sex. Emmeans tests were then performed to compare their effects across all treatments. We checked whether data distribution conformed with the expectation of a normal distribution and log‐transformed data where necessary. Experimental adults were derived from either eggs or second‐instar nymphs placed into the temperature treatments, and both sets of data were analysed in the same way. The GST results were analysed using a two‐way ANOVA, including temperature and morph effects, followed by Tukey's post hoc analysis.

SWATH quantitative analysis was performed using Skyline (19.1.0.193), an open‐source software program (https://proteome.gs.washington.edu/software/skyline). Quantitative values were based on extracted ion chromatograms (XICs) from SWATH MS2 data. To assess proteomic differences between pale‐mutant and wild‐type, the data were first normalised using the normalize_vsn function from the DEP package (v1.26.0). Principal Component Analysis (PCA) was performed using the prcomp function from factoextra (v1.0.7). Differentially expressed proteins between morphs were identified using the test_diff function of the DEP package. False discovery rate (FDR) correction of *p*‐values was performed using the fdrtools package. Findings were considered significant at an FDR‐corrected *p*‐value of 0.05. To annotate identified protein functions, homologues were sought by querying peptides against the blastp database on default parameters.

## 3 Results

### 3.1 Impact of Temperature on Colour Polymorphism

The warning colours of *Lygaeus simulans*, exposed to temperature treatments at the embryonic (egg) (Figure 1) or post‐embryonic (nymph) (Figure S3) stage, varied significantly across temperatures and morphs (wild‐type versus pale‐mutant).

Luminance increased with temperature (*F*_3,312_ = 62.60, *p* < 0.001), and the effect was much more pronounced in the pale‐mutant (Interaction: *F*_3,312_ = 32.91, *p* < 0.001; Figure 1a). Pale‐mutants had a higher luminance overall (*F*_1,312_ = 749.04, *p* < 0.001).

With respect to the Red‐Green channel, wild‐type bugs were, unsurprisingly, significantly redder than pale‐mutant bugs (*F*_1,312_ = 1691.71, *p* < 0.001; Figure 1b). All bugs were less red at higher temperatures (*F*_3,312_ = 88.14, *p* < 0.001), although this effect differed between the two morphs (interaction: *F*_3,312_ = 8.24, *p* < 0.001), with pale‐mutants changing more than wild‐types. Thus, both pale‐mutant and wild‐type bugs were redder at the lower temperature of 24°C, and pale‐mutants looked like normal wild‐type bugs to the naked eye at this temperature (Figure 1d).

The results for the Yellow‐Blue channel were similar to the luminance results. Bugs were yellower with increasing temperature (*F*_3,312_ = 68.02, *p* < 0.001; Figure 1c), pale‐mutants were yellower than wild‐types (*F*_1,312_ = 976.75, *p* < 0.001), and temperature affected pale‐mutants more than wild‐types (Interaction: *F*_3,312_ = 24.52, *p* < 0.001).

Overall, there were significant effects of both morph and temperature treatment on the warning colours, and this was consistent between egg and nymph treatments. Adults reared at 24°C had lower colour values in the Luminance and Yellow‐Blue channels, and higher values in the Red‐Green channel compared to those reared at higher temperatures, a change that could also be seen by eye (Figure 1d; Figure S3d). At and above 29°C, the colour appears to stabilise, as we found no significant differences between the 29°C and 32°C treatments in any of the colour channels (*p* > 0.05 for all post hoc tests, Figure 1a–c).

### 3.2 Effect of Temperature on Weight and Body Size

Adult weight was affected by rearing temperature (Figure 2; Figure S4). Bug weight significantly increased with increasing rearing temperature (*F*_3,810_ = 237.61, *p* < 0.001; Figure 2a,b), an effect that was more pronounced in wild‐types than in the pale‐mutants at 24°C (Interaction: *F*_3,810_ = 15.28, *p* < 0.001). Females were heavier than males (*F*_1,810_ = 347.73, *p* < 0.001), and the pale‐mutants were heavier than the wild‐types at 24°C (*F*_1,810_ = 20.99, *p* < 0.001).

**FIGURE 2:**
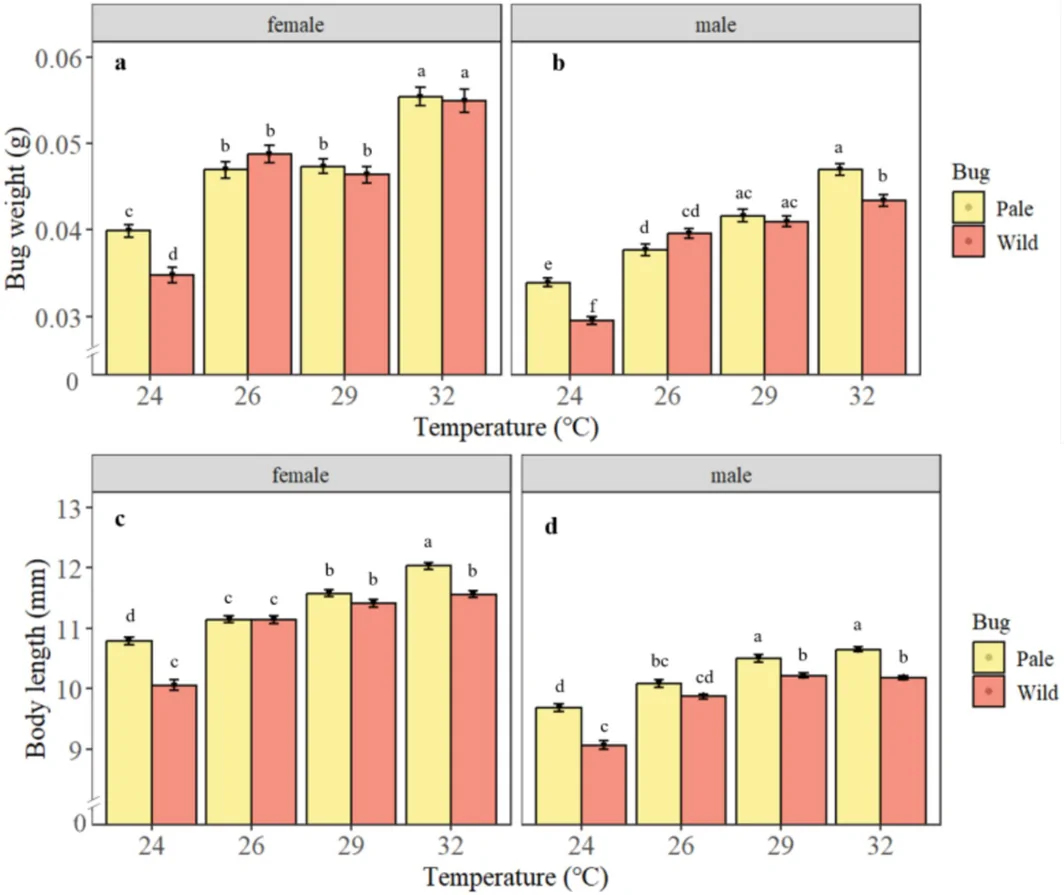
The effect of temperature on adult weight (*N* = 826) and body length (*N* = 808). (a) female weight; (b) male weight; (c) female body length; (d) male body length. Pale‐mutants are shown in yellow and wild‐types are shown in red. Results shown in this figure are from bugs transferred to the temperature treatments at the egg stage. Bars represent means of weight or body length ± SEs.

The results for body length were broadly the same (Figure 3; Figure S4). Bugs reared at a higher temperature were larger (*F*_3,792_ = 310.93, *p* < 0.001; Figure 3c,d), with a significant interaction between morph and temperature (*F*_3,792_ = 19.80, *p* < 0.001). Females were larger than males (*F*_1,792_ = 1808.20, *p* < 0.001), and pale‐mutant morphs were larger than wild‐types (*F*_1,792_ = 205.72, *p* < 0.001).

**FIGURE 3:**
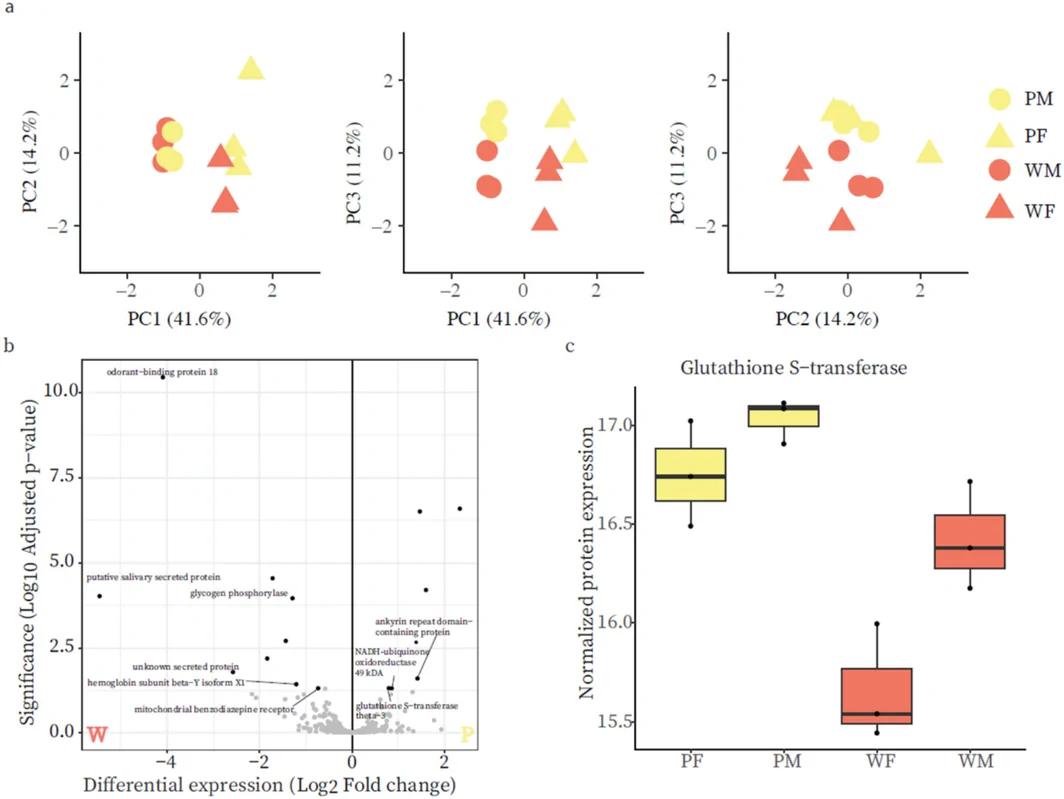
SWATH‐MS analysis (*N* = 12, 3 per sex per morph) of pale‐mutant and wild‐type bugs (a) Principal component analysis (PCA) of both morphs and sexes PC1 against PC2 (left) is separated by sex, and PC2 against PC3 (right) is separated by morph. PC1 against PC3 (middle) is separated by morph, with great separation observed between morphs. (b) Volcano plot of identified proteins from whole‐body proteomes. Significantly differentially expressed proteins are marked in black. Proteins for which a characterised homologue was identified using BLAST are labelled. A positive log_2_ fold change indicates proteins upregulated in the pale‐mutant compared to the wild‐type morph. (c) *Glutathione S‐transferase* expression between morphs and sex. Pale‐mutants are shown in yellow and wild‐types are shown in red. PF and PM in *X*‐axis represent pale‐mutant females and males, respectively. WF and WM in *X*‐axis mean wild‐type females and males, respectively.

### 3.3 Proteomics Analysis

SWATH‐MS analysis identified the expression of 709 proteins. PCA revealed substantial proteomic differences between sexes and morphs (Figure 4a). PC1 explained 41.6% of the variance and separated the data along the sex axis. PC3 explained 11% of the variance and separated the data by morph. Finally, 15 significantly differentially expressed proteins were identified between the colour morphs (Figure 3b; Table S1). The identified proteins are primarily involved in metabolic processes (e.g., Glycogen phosphorylase), oxidative stress response (e.g., *Glutathione S‐transferase* (GST)), and chemical communication (e.g., Salivary secreted proteins and Odorant‐binding proteins). Notably, GST attracted our particular interest due to its multiple roles in detoxification and oxidative stress regulation in insects (Pavlidi et al. 2018). We found that GST levels were significantly upregulated in pale‐mutants compared to the wild‐types (log2 fold change: 0.79, *p* = 0.048, Figure 3c).

**FIGURE 4:**
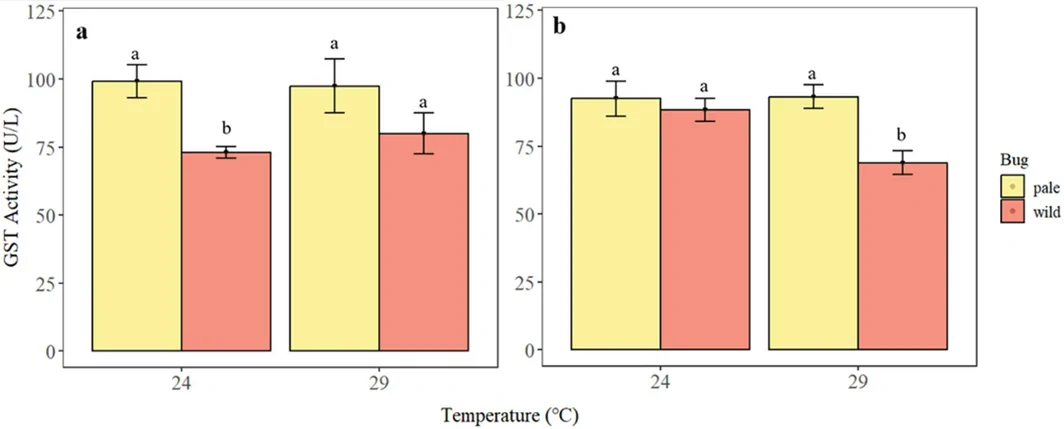
GST enzyme activity in *Lygaeus simulans* (*N* = 72, 9 per temperature, morph and sex combination). (a) Female and (b) Males. Pale‐mutants are shown in yellow and wild‐types are shown in red. Bars represent means of GST activity± SEs. The letters represent significance from post hoc Tukey's tests.

### 3.4 GST Enzymatic Activity Assays

GST activity in bugs raised under two temperatures was analysed to explore the effects of temperature on GST and its role in the variation. We found that pale‐mutant and wild‐type individuals had different GST activity at 29°C (Figure 4), which matched the results presented above from a whole‐proteome approach, but the pattern was absent at 24°C in wild‐type males.

In more detail, female GST activity was higher in pale‐mutants (*F*_1,32_ = 9.48, *p* = 0.0042), and this difference was not affected by temperature (*F*_1,32_ = 0.0007, *p* = 0.91). For males, at 24°C there was no difference between pale‐mutant and wild‐type GST activity, whilst at 29°C the pattern seen in females was observed, with pale‐mutant individuals having higher GST activity than wild‐types. Overall, pale‐mutant males had higher GST activity (*F*_1,32_ = 8.63, *p* = 0.006), but temperature was marginally significant as a main effect (*F*_1,32_ = 4.09, *p* = 0.052).

## 4 Discussion

### 4.1 Effects of Temperature

Our main findings are that *L. simulans* colouration is strongly temperature‐dependent. Colour shifts occurred regardless of whether individuals were exposed to temperature treatments at the embryonic (egg) or post‐embryonic (nymph) stage, indicating that thermal plasticity in warning signals can occur after hatching. While many studies have shown temperature‐sensitive variation in melanin‐based patterns in insects (Delhey 2018, 2019), temperature sensitivity in red colouration is much more unusual. Moreover, very few studies have reported temperature‐sensitive colour mutations, where the penetrance or expression of the mutation varies with temperature. Known examples include the tachinid fly *Exorista japonica*, in which the eyes of a red mutant become redder at low temperatures and lighter at high temperatures, whereas the wild type remained brown at all temperatures (Ichiki et al. 2007). This pattern parallels our findings in *L. simulans* that both the pale mutant and wild type became redder at lower temperatures. Although clear differences remained in the colour analysis at lower temperatures, it became difficult to distinguish pale‐mutant and wild‐type bugs by eye when they were reared at 24°C, while the two are visually distinct when reared at 29°C (Figure 1d).

Since we found that *L. simulans* eggs failed to hatch when the parent bugs were reared at 24°C (Zhao, pers. Observation), 24°C may either be stressful for them or sub‐optimal for reproduction. A closely related species, *L. equestris* , enters adult reproductive diapause over the winter (Shuker et al. 2006), which is seen as a reduction in reproductive activity even at temperatures of 25°C (Sillén‐Tullberg 1981). In contrast, while 32°C is likely to be at the high end of the natural temperature range they encounter (Heyden and Dioli 2018), the bugs could still successfully breed at this temperature (Zhao, pers. Observation), which suggests that it is not overly stressful for them. While field studies on *L. simulans* remain to be done, the data from both *L. equestris* and also from *L. simulans* in the laboratory suggest that the reductions in reproductive behaviour at 24°C and below are ecologically relevant with respect to changes in temperature as summer moves to autumn across the species ranges in the Palearctic (Heyden and Dioli 2018).

### 4.2 Trade‐Offs of Colour Variation

Increased red coloration at low temperatures may enhance distinctiveness against natural backgrounds and promote recognition by predators. Birds have the capability to distinguish variation in the redness of aposematic warning signals (Lindstedt et al. 2011), and in some systems brighter colours indicate greater toxicity (i.e., honest signalling; Blount et al. 2012; Stevens and Ruxton 2012). Therefore, the redder colour of bugs at low temperatures could improve defence by advertising their ‘toxicity’ during periods when alternative insect prey is scarcer. However, at present very little is known about colour variation in wild *L. simulans* , both geographically or across the seasons, or indeed its effects on predation risk, so this hypothesis would require further study.

The bugs not only became redder at lower temperatures, but also became smaller and had a lower survival ratio (Figure S5) than those reared at higher temperatures. Although temperature has long been considered a crucial environmental cue for insect development (Chown and Nicolson 2004), given that both morphs also showed colour changes across treatments, we cannot rule out a possible cost of this colour change itself. Colouration switch is known to be physiologically costly in moths (Ethier et al. 2015; Ma et al. 2008; Lee and Wilson 2006). Many pigments such as pteridines and melanins are synthesised de novo. Their precursors are essential amino acids (Talal et al. 2020). Synthesis of pigments therefore results in resource competition of these amino acids in vivo (Lindstedt 2008). Another possible explanation is that the oxidative state at lower temperatures caused the trade‐offs. Herbivores need to balance their oxidative state as they are often exposed to toxic plant allelochemicals (Ahmad 1992). Thus, the ability of *L. simulans* to sequester toxic cardenolides (Bramer et al. 2015) is anticipated to result in a finely tuned oxidative state system in vivo. Antioxidants act in detoxification, as well as warning colour synthesis in insects (Ito and Prota 1977; Blount et al. 2023). It is hypothesised that insects accumulate antioxidants to balance their oxidative state to protect themselves (Blount et al. 2009, 2023), but once antioxidants are invested in a colour synthesis pathway they cannot be used for other purposes, leading to oxidative stress and possible trade‐offs with life‐history traits and other components of fitness (Blount et al. 2009). Recent work in *Oncopeltus fasciatus* has shown that sequestration of toxic cardenolides reduces total glutathione levels and results in duller warning coloration, supporting the idea of a resource allocation trade‐off between oxidative defence and pigment investment (Heyworth et al. 2023).

### 4.3 Protein Function

Differences between colour morphs are often associated with concurrent changes to suites of behavioural and life history traits (Nishikawa et al. 2015), as well as gene expression (Galarza et al. 2019). For example, in the wood tiger moth, 302 differentially expressed genes were identified through RNA sequencing between albino and coloured caterpillars (Galarza 2021). In this study, we used proteomics to explore protein differences between pale‐mutant and wild‐type. Although the comparison is limited by the small sample size, we tentatively identified 15 differentially expressed proteins in our whole‐proteome comparison (Table S1). These are involved in metabolism, energy, oxidative state and chemical communication. For example, glycogen phosphorylase is associated with carbohydrate conversion in insects (Zeng et al. 2020). Salivary secreted proteins are secreted by plant‐feeding insects in food probing and the ingestion process (Huang et al. 2018). Odorant‐binding proteins 18 (OBPs) play an important role in insect chemical communication, where OBPs transport pheromones or odours to odorant receptors (Rong et al. 2024; Xu et al. 2009).

We also found differential expression of Glutathione‐S‐transferase (GST) between wild‐type and pale‐mutant morphs with a marginal statistical significance (*p* = 0.048). This attracted our attention due to its multifunctional roles in insects, such as detoxification (Pavlidi et al. 2018), a function that has been reported in moths (Li et al. 2022), beetles (Song et al. 2022), and aphids (Lin et al. 2022). Some limited evidence suggests that GSTs may contribute to the synthesis or transport of yellow and red pigments such as pheomelanin and flavonoids (Carletti et al. 2014; Arai et al. 2017). Insects also produce extra reactive oxygen species (ROS) in response to stress (e.g., heat), where the GST enzyme is activated (González‐Tokman et al. 2024). We found that temperature has an association with GST level, although we did not find a simple linear relationship between temperature and GST in *L. simulans* , as the effect varied between males and females and between morphs. Pale‐mutant bugs had consistent GST activity regardless of temperature or sex. Wild‐type females also had consistent GST activity, but males had increased GST activity, similar to that of pale‐mutants at 24°C.

The association between temperature and GST activity has been previously explored in insects. For instance, in the mite *Panonychus citri* (McGregor), higher GST activity was found in individuals reared at 35°C than at the control temperature (25°C; Yang et al. 2010). Similarly, Dampc et al. (2020) found an increase in GST activity in the aphid *Aphis pomi* at high temperatures. However, male wasps of *Aphelinus asychis* had irregular GST activity at higher temperatures, whereas GST activity in females was positively associated with temperature, indicating sex differences in enzyme activity (Liu et al. 2022). Taken together, these results highlight that GST activity can be shaped by multiple interacting factors, including sex and environment, but much remains to be done in *L. simulans* and more generally.

## 5 Conclusion

We found that aposematic colouration in *L. simulans* is temperature dependent, and this is more pronounced in the pale‐mutant, which exhibits the wild‐type colouration when reared at 24°C. Furthermore, we found proteomic differences between the pale‐mutant and the wild‐type bugs, and identified 15 differentially expressed proteins involved in metabolism, energy, and oxidative state. While there is increasing evidence that selective pressures impact aposematic warning signals at the population level, the exact mechanisms linking the underlying genes to patterns of pigment production and deployment still require further study. Our findings are a first attempt in understanding the evolution and maintenance of colour polymorphism in aposematic species.

## Author Contributions

**Jiajia Zhao:** formal analysis (equal), visualization (equal), writing – original draft (equal), writing – review and editing (equal). **Daniella Black:** formal analysis (equal), software (equal), visualization (equal), writing – review and editing (equal). **Madingley M. Abbs:** writing – review and editing (equal). **Terry K. Smith:** software (equal), writing – review and editing (equal). **Sally L. Shirran:** methodology (equal), software (equal), writing – review and editing (equal). **Silvia A. Synowsky:** methodology (equal), software (equal), writing – review and editing (equal). **Hannah M. Rowland:** writing – review and editing (equal). **Astrid T. Groot:** writing – review and editing (equal). **David M. Shuker:** supervision (equal), writing – review and editing (equal). **Emily R. Burdfield‐Steel:** supervision (equal), visualization (equal), writing – review and editing (equal).

## Funding

This work was supported by China Scholarship Council (CSC) Scholarship, The Laidlaw Foundation 2026, The Wellcome Trust, 094476/Z/10/Z, BBSRC East Bio Doctoral Training Partnership.

## Conflicts of Interest

The authors declare no conflicts of interest.

## Supporting information


**Data S1:** Supplementary excel.

**Data S2:** Consequences of temperature on bug survival.

**Figure S1:** Aposematic colour regions of interest in *Lygaeus simulans* Left: wild‐type: Right: pale‐mutant. The lower letters a and b denote areas used for colour analysis.
**Figure S2:** Measured body length of (a) male (b) female *L. simulans* (individual shown is a pale‐mutant individual).
**Figure S3:** Warning colour signals of adults reared at different temperatures (*N* = 320). Warning colour was estimated by (a) Luminance, (b) Red and Green opponent channels and (c) Yellow and Blue opponent channels. Pale‐mutantss are shown in yellow and wild‐types are shown in red. *X*‐axis represents different temperatures (24°C, 26°C, 29°C and 32°C). (d) Aposematic colour of bug living in different temperatures. The adults in the above figure were transferred to the temperature treatments as second‐instar nymphs. Bars represent means of colour value ± SEs. Letters represent significant differences from Tukey's post hoc tests.
**Figure S4:** The effect of temperature on adult weight (N = 892) and body length (N = 906). (a) female weight; (b) male weight; (c) female body length; (d) male body length. Pale‐mutants are shown in yellow and wild‐types are shown in red. The adults in the above figure were transferred to the temperature treatments as second‐instar nymphs. Bars represent means of weight or body length ± SEs. In the egg treatment, wild‐type bugs living at 24°C had lower survival than those living at 29°C (z = −5.35, p < 0.001), while we didn't find this pattern in pale‐mutant (z = −1.18, p = 0.94). In the nymph treatment, pale‐mutant bugs living at 24°C had lower survival than those living at 29°C (z = −3.543, p = 0.009), while the wild‐type didn't show the pattern (z = −0.367, p = 1).
**Figure S5:** The survival ratio of adults on different temperatures (*N* = 80). Pale‐mutants are shown in yellow and wild‐types are shown in red. The left plot: the adults were reared at the temperatures from egg stage; The right plot: the adults were reared at the temperatures from nymph stage. Bars represent means of ratio ± SEs.
**Table S1:** Differentially expressed proteins between pale‐mutant and wild‐type in *L. simulans* . Proteins were considered differentially expressed between colour morphs at an FDR corrected *p*‐value of 0.05 (using *test_diff* from DEP package). A negative log2 fold change represents enrichment in the wild‐type morph and a positive log2 fold change represents enrichment in the pale‐mutant. To predict the function and identity of differentially expressed proteins, the protein sequence was queried with BLASTP, the accession name and alignment statistics for the top hit is listed.

## Data Availability

Additional supporting information can be found in the Methods S1 and Results S1. The mass spectrometry proteomics data have been deposited in the ProteomeXchange Consortium via the PRIDE partner repository with the dataset identifier PDX0666666. Colour raw data can be found in figshare (https://figshare.com/s/659d20686840e7d2d725).
