# Supplementary material for: Temperature‐Dependent Warning Colour Variation in an Aposematic Insect
Source: Ecol Evol. 2026 Nov 10;16(11):e73683. doi: 10.1002/ece3.73683 (PMC13749155; doi:10.1002/ece3.73683)
Supplement: Supplementary file 1 [file ECE3-16-e73683-s001.docx]

Figure S1. Aposematic colour regions of interest in *Lygaeus simulans* Left: wild-type: Right: pale-mutant. The lower letters a and b denote areas used for colour analysis.

Figure S2. Measured body length of (a) male (b) female *L. simulans* (individual shown is a pale-mutant individual).

Figure S3. Warning colour signals of adults reared at different temperatures (*N* = 320). Warning colour was estimated by (a) Luminance, (b) Red and Green opponent channels and (c) Yellow and Blue opponent channels. Pale-mutantss are shown in yellow and wild-types are shown in red. X-axis represents different temperatures (24 °C, 26°C, 29 °C and 32°C). (d) Aposematic colour of bug living in different temperatures. The adults in the above figure were transferred to the temperature treatments as second-instar nymphs. Bars represent means of colour value ± SEs. Letters represent significant differences from Tukey’s post-hoc tests.

Figure S4. The effect of temperature on adult weight (*N*=892) and body length (*N*=906). (a) female weight; (b) male weight; (c) female body length; (d) male body length. Pale-mutants are shown in yellow and wild-types are shown in red. The adults in the above figure were transferred to the temperature treatments as second-instar nymphs. Bars represent means of weight or body length ± SEs.

In the egg treatment, wild-type bugs living at 24 °C had lower survival than those living at 29 °C (z =-5.35, *p*<0.001), while we didn’t find this pattern in pale-mutant (z =-1.18, *p*= 0.94). In the nymph treatment, pale-mutant bugs living at 24 °C had lower survival than those living at 29 °C (*z* =-3.543, *p*=0.009), while the wild-type didn’t show the pattern (*z* =-0.367, *p*= 1).

Figure S5. The survival ratio of adults on different temperatures (*N*=80). Pale-mutants are shown in yellow and wild-types are shown in red. The left plot: the adults were reared at the temperatures from egg stage; The right plot: the adults were reared at the temperatures from nymph stage. Bars represent means of ratio ± SEs.

Table S1: Differentially expressed proteins between pale-mutant and wild-type in *L. simulans*. Proteins were considered differentially expressed between colour morphs at an FDR corrected *p*-value of 0.05 (using *test_diff* from DEP package). A negative log2 fold change represents enrichment in the wild-type morph and a positive log2 fold change represents enrichment in the pale-mutant. To predict the function and identity of differentially expressed proteins, the protein sequence was queried with BLASTP, the accession name and alignment statistics for the top hit is listed.

| Differential expression analysis | | BLASTP alignment | | | | | | |
| --- | --- | --- | --- | --- | --- | --- | --- | --- |
| log2 Fold change | Adjusted  *p*-value | Protein accession | Protein description | Identity (%) | Alignment length | Mismatches | Gap opens | e-value |
| -5.46 | 9.55E-05 | XGD09057.1 | putative salivary secreted protein *Riptortus pedestris* | 58.065 | 124 | 50 | 2 | 1.41E-44 |
| -4.09 | 3.60E-11 | AXB87333.1 | odorant-binding protein 18 *Tropidothorax elegans* | 36.216 | 185 | 118 | 0 | 1.36E-38 |
| -2.57 | 0.0162 | BAN20133.1 | unknown secreted protein *Riptortus pedestris* | 41.379 | 145 | 84 | 1 | 8.50E-26 |
| -1.83 | 0.00647 | CAH1403219.1 | unnamed protein product *Nezara viridula* | 35.938 | 128 | 73 | 5 | 1.30E-12 |
| -1.72 | 2.85E-05 | BAN20939.1 | unkown protein *Riptortus pedestris* | 66.341 | 205 | 69 | 0 | 6.00E-88 |
| -1.43 | 0.00197 | BAN20939.1 | unkown protein *Riptortus pedestris* | 53.425 | 219 | 102 | 0 | 9.38E-71 |
| -1.29 | 0.00011 | XP_014292615.1 | glycogen phosphorylase *Halyomorpha halys* | 85.595 | 840 | 120 | 1 | 0 |
| -1.21 | 0.0368 | XP_014283476.1 | hemoglobin subunit beta-Y isoform X1 *Halyomorpha halys* | 38.356 | 146 | 90 | 0 | 6.10E-29 |
| -0.733 | 0.0482 | BAN20198.1 | mitochondrial benzodiazepine receptor, putative *Riptortus pedestris* | 62.5 | 168 | 60 | 2 | 8.90E-67 |
| 0.794 | 0.0481 | XP_014289737.1 | glutathione S-transferase theta-3 *Halyomorpha halys* | 63.182 | 220 | 81 | 0 | 2.26E-99 |
| 0.856 | 0.0485 | XP_014274971.1 | NADH-ubiquinone oxidoreductase 49 kDa subunit *Halyomorpha halys* | 84.582 | 467 | 71 | 1 | 0 |
| 1.39 | 0.00216 | CAH1398336.1 | unnamed protein product *Nezara viridula* | 43.333 | 360 | 148 | 15 | 5.09E-14 |
| 1.41 | 0.0249 | XP_064650397.1 | ankyrin repeat domain-containing protein 50-like isoform X2 *Lineus longissimus* | 34.156 | 243 | 148 | 4 | 9.96E-25 |
| 1.47 | 3.14E-07 | XP_065925457.1 | uncharacterized protein *Magallana gigas* | 37.5 | 160 | 86 | 8 | 1.25E-13 |
| 2.33 | 2.59E-07 | KAI8493609.1 | hypothetical protein Bbelb_285300 *Branchiostoma belcheri* | 33.456 | 272 | 142 | 6 | 2.55E-22 |
