# Supplementary material for: Temperature‐Dependent Warning Colour Variation in an Aposematic Insect
Source: Ecol Evol. 2026 Nov 10;16(11):e73683. doi: 10.1002/ece3.73683 (PMC13749155; doi:10.1002/ece3.73683)
Supplement: Supplementary file 2 [file ECE3-16-e73683-s002.docx]

**Consequences of temperature on bug survival**

Bugs were transferred to the temperatures either as eggs or second-instar nymphs (*n* =5, boxes of ~25 individuals per treatment, morph, and stage). We checked the boxes daily for newly moulted adults, and the number of individuals per box that survived to adulthood was recorded.

Survival data was analysed using a generalized linear model with a binomial error distribution and logit link. The response variable was the number of individuals that survived or died in each group. The model included morph, temperature, as well as their interaction. The effect of temperature was calculated using the emmeans. Data analysis and visualization were performed using R studio.

**Proteomics analysis**

*Bugs for* *proteomics*

To assess the proteome of pale-mutant and wild-type *L. simulans* morphs, SWATH-MS analysis was performed with 3x biological replicates per sex/morph group on the whole body of reproductively mature virgin adults. To collect required bugs, approximately 10 fifth-instar nymphs of both morphs were kept in nymph box and were provided with sunflower seeds, a cotton-plugged water tube and cotton wool as habitat. Newly eclosed adults were checked daily and separated into individual boxes. These adults were housed in the incubator for between 7 and 9 days before experimentation.

*Protein extraction and measurements*

To obtain a homogenate of each sample, bugs were frozen for 20 min and individually homogenized using the insects (light and hard) Bullet Blender® homogenization protocol green bead lysis kit. Following the dry grinding step 350 μl of 5M urea (invitrogen, UltrapureTM purity > 99%) was used as a homogenization buffer. The bullet blender was cooled throughout using dry ice. Samples were centrifuged for 5 min, 13,000 rpm, and the supernatant transferred to a protein low-bind tube, snap frozen in liquid nitrogen and stored at –80 °C before further processing.

Proteins were extracted using 8M urea/2% Sodium Deoxycholate in 50mM Ammonium Bicarbonate. The protein concentration of each sample was measured using the PierceTM BCA protein assay kit according to manufacturer’s instructions. 30 μg of each sample were reduced with TCEP (5 mM) at 30 °C for 45 min followed by alkylation with iodoacetamide (10 mM) in the dark at room temperature for 30 min. Reaction was quenched with 20mM DTT. Each sample was digested with trypsin (1:50; protease: protein) overnight at 30 °C.

Peptides were desalted by C18 and then dried. Peptides were then resuspended to a concentration of 1 ug/ul in loading buffer (2% acetonitrile, 0.05% TFA). SWATH acquisition was performed on individual samples (SWATH/MS). In addition, a pool of all samples was prepared and subjected to nanoLC MS/MS analysis (IDA-LC-MS/MS).

To create an IDA library of protein identifications for interrogation of SWATH data sets, a combined sample from all morphs, sexes and replicates (1 μg) was injected onto a reverse-phase trap (Acclaim Pepmap 100 μm × 2 cm, Thermo Fisher) for pre-concentration and desalting with loading buffer, at 5 μL/min for 10 min on a nanoLC-MS system (Eksigent nanoLC AS-2/2Dplus system coupled to a Triple TOF 5600+ mass spectrometer, both Sciex). The peptide trap was then switched into line with the analytical column (Acclaim Pepmap RSLC 75 μm × 15 cm, Thermo Fisher). The mobile phase was composed of 2% acetonitrile and 0.1% formic acid in water (A) as well as 0.1% formic acid in acetonitrile (B). Peptides were eluted from the column at a flow rate of 300 nl/min using a linear solvent gradient: linear 2–20% of buffer B over 90 min, linear 20–40% of buffer B for 30 min, linear 40–98% of buffer B for 10 min, isocratic 98% of buffer B for 5 min, linear 98-2% of buffer B for 2.5 min and isocratic 2% solvent buffer B for 12.5 min. The mass spectrometer was operated in IDA top 20 positive ion mode, with 250 and 150 ms acquisition time for the MS1 (*m/z* 400–1250) and MS2 (*m/z* 230–1800) scans respectively, and 15 s dynamic exclusion. Rolling collision energy with a collision energy spread of 5 eV was used for fragmentation. The mass spectrometric files were converted using Sciex converter for analysis. These files were searched using an in-house database with trypsin as the cleavage enzyme and carbamidomethylation as a fixed modification of cysteines. The database was generated by converting transcriptome data from the closely related *Lygaeus* species, *L. equestris* and *L. turcicus*, to cDNA libraries (Johnson et al, 2018). The *L. equestris* and *L. turcicus* cDNA libraries were then translated in all 6 possible reading frames to create one protein database. The iRT peptides were added to this database.

*Data independent acquisition (SWATH)*

To analyse samples with SWATH-MS, 1 μg of each sample was injected onto the same LCMS set up and gradient as above, however data acquisition was done in SWATH mode. The method uses 100 variable windows width with a 1 Da overlap as developed and optimized by Sciex on Plasma. Each window has a dwell time of 150 ms to cover the mass range of 400–1250 *m/z* in TOF-MS mode and MS/MS data is acquired over a range of 230–1800 *m/z* with high sensitivity setting and a dwell time of 35 ms, resulting in a cycle time of 3.7 s. The collision energy for each window was set using the collision energy of a 2+ ion centred in the middle of the window with a spread of 5 eV.

**Reference**

Johnson, K.P., Dietrich, C.H., Friedrich, F., Beutel, R.G., et al., (2018) Phylogenomics and the evolution of hemipteroid insects. *Proceedings of the National Academy of Sciences of the USA*, 115, 12775-12780.
